# A Review on Lymphatic Uptake of Large Therapeutic Proteins after Subcutaneous Injection

**DOI:** 10.1007/s11095-026-04171-8

**Published:** 2026-09-03

**Authors:** Ehsan Rahimi, Xiaoxu Zhong, Chenji Li, Xin Zhang, Galen Huaiqiu Shi, Arezoo M. Ardekani

**Affiliations:** 1 School of Mechanical Engineering, Purdue University, West Lafayette, IN 47907 USA; 2 Eli Lilly and Company, Indianapolis, IN 46225 USA

**Keywords:** lymphatic uptake, large therapeutic proteins, pharmacokinetics, subcutaneous administration

## Abstract

Subcutaneous (SC) administration has emerged as a promising route for delivering large therapeutic proteins, offering advantages such as improved patient convenience and reduced healthcare costs. However, the mechanisms that regulate the uptake of these macromolecules are not yet fully understood. A recent surge in computational and experimental studies has systematically analyzed the transport of large proteins from the SC injection site to the lymphatic system, advancing our understanding of how tissue environments and injection parameters affect lymphatic uptake kinetics. This review summarizes the current literature on factors affecting the lymphatic uptake of subcutaneously administered large therapeutic proteins. First, we outline the transport mechanisms of therapeutic proteins from the local SC injection site to initial lymphatics. Then we analyze the impact of injection site, tissue properties, device selection, and molecular characteristics, on lymphatic uptake kinetics and pharmacokinetics (PK). Finally, we evaluate how recent modeling and experimental advancements have transformed our mechanistic understanding of SC delivery.

## Introduction

Therapeutic proteins have achieved significant success in the treatment of a wide range of diseases, including cancer, diabetes, genetic disorders, and neurodegenerative disorders. Among these, large therapeutic proteins, such as monoclonal antibodies (mAbs), constitute a substantial portion of the market, accounting for approximately half of all sales in Europe and the USA [1]. These large proteins are unsuitable for oral administration because they undergo denaturation in the acidic stomach environment, are susceptible to proteolytic degradation in the gastrointestinal tract, and exhibit limited diffusion across the gastrointestinal epithelium [2]. Consequently, parenteral routes, including intramuscular, SC, and intravenous, are preferred. The SC route is particularly prevalent, with self-administration using autoinjectors (AIs) becoming increasingly common [3–5]. However, compared to intravenous delivery, SC administration is associated with lower bioavailability due to pre-systemic degradation.

**Fig. 1:**
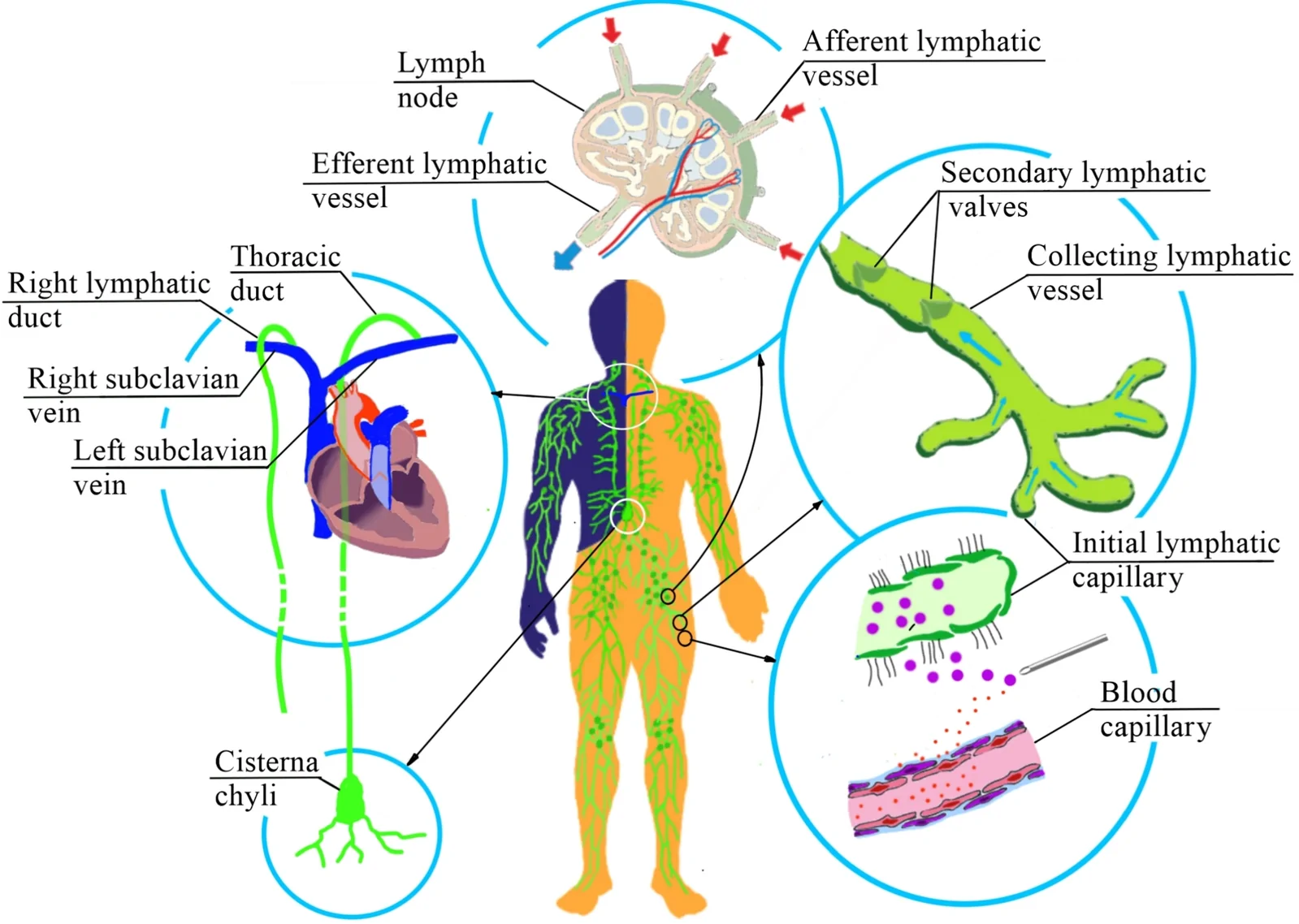
Schematic of the lymphatic system. Reproduced with permission from Computers in Biology and Medicine [10].

The route by which large therapeutic proteins reach the systemic circulation depends heavily on their molecular weight (MW). While compounds below 40 kDa utilize both blood and lymphatic pathways (Fig. 1), molecules above 40 kDa are almost exclusively taken up by lymphatic capillaries [6]. Most subcutaneously administered therapeutic mAbs (∼ 150 kDa) require 2 to 8 days to reach the peak concentration in the plasma [7, 8]. This delay is primarily attributed to slow interstitial transport from the injection depot to the initial lymphatics [9, 10], given that subsequent transit through the lymphatic system into the blood circulation is rapid. Recent sensitivity analyses further emphasize that this lymphatic uptake rate can significantly affect both overall bioavailability and the time to peak plasma concentration [11]. Despite growing interest in how formulation, device, and injection site variations impact the bioavailability of large therapeutic proteins, a complete mechanistic understanding remains elusive due to the complex interplay among delivery parameters, tissue mechanics, and biotherapeutic properties.

While previous reviews have detailed the PK of subcutaneously administered proteins and lymphatic structure [6, 7, 9, 12, 13], the understanding of specific determinants governing the initial lymphatic uptake rate remains limited. Transport within the SC tissue is driven by convection and diffusion, with convection heavily influenced by tissue pressure and deformation. Additionally, these molecules must traverse the SC layer to reach the initial lymphatic vessels in the dermis. This transport process is affected by the tissue properties, drug plume morphology, and therapeutic-protein molecular characteristics; and the quantitative understanding of this injection-transport-absorption process requires a clearer elucidation. It is worth mentioning that although the previous study [14] summarized the impact of blood flow, injection site, injection technique, concentration, and volume on subcutaneously administered insulin, insulin monomers and dimers are readily absorbed by blood capillaries. Therefore, the transport observations for insulin are not directly applicable to the large therapeutic proteins evaluated here.

Because the lymphatic uptake of large therapeutic proteins following SC administration involves multiple factors that impact multi-scale transport processes and subsequent PK, a comprehensive synthesis is needed. In this review, we focus on lymphatic uptake and synthesize current evidence regarding its key determinants, including injection location, physiological transport conditions (e.g., lymphatic flow and vessel functionality), and protein attributes such as MW and charge. Furthermore, we discuss the impact of the lymphatic uptake rate on the systemic PK of these macromolecular proteins. We start by discussing the transport of these large therapeutic proteins from the injection location to the systemic circulation through the lymphatic system. Then, we examine the potential factors and mechanisms that affect lymphatic uptake and bioavailability. Next, we discuss key experimental and computational studies on the lymphatic uptake of large proteins, focusing on recent advancements in the field. Finally, we highlight the existing gaps and limitations in the literature concerning subcutaneous delivery and lymphatic uptake, pointing out areas where further research is needed.

**Fig. 2:**
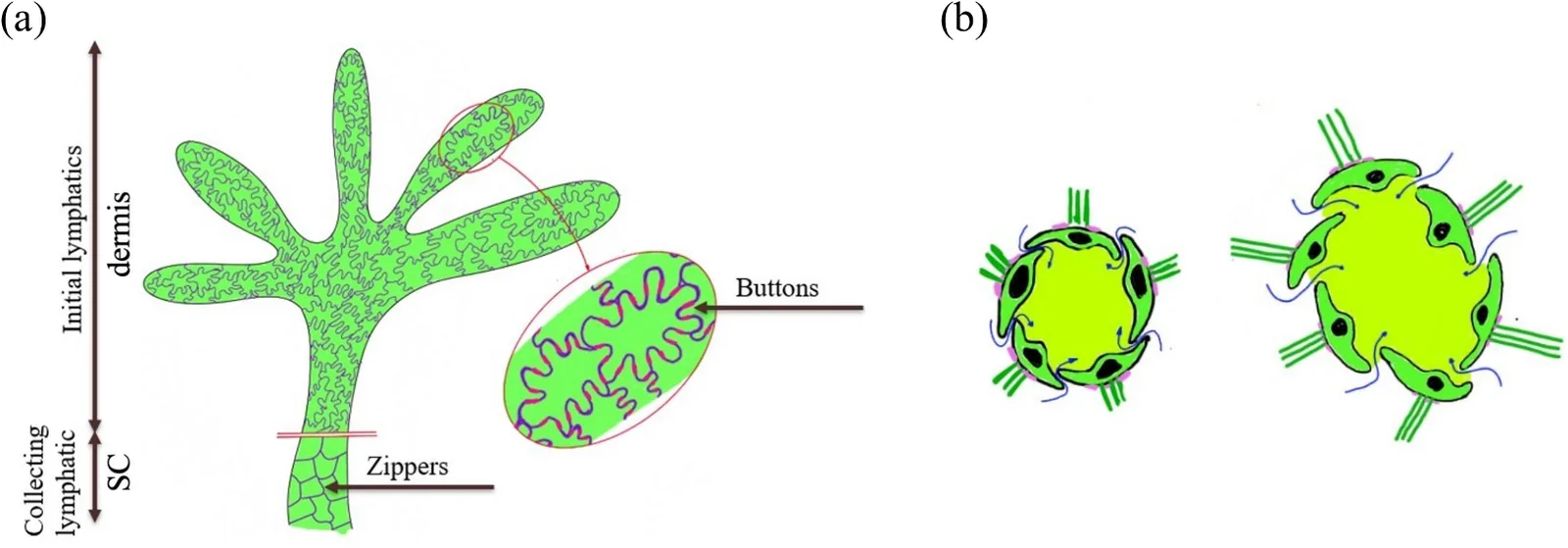
(**a**) Endothelial junctions at the membrane of lymphatic capillaries (**b**) initial lymphatics in initial and deformed configurations. Reproduced with permission from Computers in Biology and Medicine [10].

Note that this review focuses on large therapeutic proteins, among which mAbs represent the most extensively studied class and serve as our primary example. Literature databases including PubMed and Google Scholar are searched using targeted keywords such as subcutaneous administration, lymphatic uptake, initial lymphatics, therapeutic proteins, pharmacokinetics, autoinjectors, prefilled syringes, injection site, compartment model, and mathematical modeling. The quality and mechanistic accuracy of the collected literature are critically evaluated through consultation with computational and experimental experts. The collected experimental and computational findings serve to summarize recent state-of-the-art modeling advancements for subcutaneously administered large therapeutic proteins while establishing a comprehensive foundation for future high-fidelity computational frameworks in this field. Many of the principles regarding delivery parameters, injection site, and biotherapeutic characteristics apply broadly to other biologic agents [6, 15]. Unless otherwise noted, our discussion pertains to human physiology, animal studies are cited only where human data are unavailable or where preclinical models have been used to elucidate transport mechanisms.

## Transport of Biotherapeutics after SC Administration

Following SC delivery, large therapeutic proteins must travel through the extracellular matrix (ECM) to reach the initial lymphatics. Diffusion in the interstitial space is significantly hindered compared to free solution, with effective diffusion coefficients reduced by 20% to 90%. This impedance is a function of the density and orientation of collagen fibers, protein size, and ionic interactions with the ECM [16]. Notably, the negative charge of glycosaminoglycan chains in the ECM attracts counter-ions to maintain electroneutrality, leading to high interstitial osmotic pressure. This environment influences fluid balance and retards the movement of proteins, particularly those with high surface charge or strong hydrophobic affinities, often leading to reduced bioavailability [13, 17].

A critical factor in lymphatic uptake is the spatial distribution of initial lymphatics. While the SC layer primarily comprises adipocytes and connective tissue for metabolic storage and protection, the dermis layer has a dense network of blood and lymphatic capillaries [18–20]. Because the lymphatic density in the dermis is significantly higher than that in the SC layer, most therapeutic proteins must migrate through the SC ECM to reach these dermal drainage sites.

Initial lymphatics are blind-ended terminal structures (Fig. 2a) with diameters ranging from 10 to 70 μ m [13, 21–24]. Unlike the continuous basement membranes of blood capillaries, the endothelial cell membranes of initial lymphatics feature discontinuous, button-like junctions [25]. These junctions act as specialized primary valves that cycle between open and closed states in response to mechanical tissue deformation (Fig. 2b). This unique mechanical architecture facilitates the uptake of large molecules, a process that has been characterized extensively through various numerical modeling frameworks [10, 26–30].

Once therapeutic proteins enter the initial lymphatics, they are transported through collecting lymphatics and lymph nodes before reaching the systemic circulation [12]. Collecting lymphatics have larger diameters (150-300 μm), and the junctions between endothelial cells in collecting lymphatics are tight and zipper-like, as shown in Fig. 2a. Unlike initial lymphatics, collecting lymphatics contain contractile muscle cells in their walls, which assist in pumping lymph fluid forward. Unidirectional flow is maintained within collecting lymphatics, which contain one-way flap valves that open and close in response to local pressure gradients [12].

Lymph nodes serve as both immunological filters and fluid regulators [22, 31]. The dense internal architecture of the lymph nodes adds significant hydraulic resistance to the flow [32, 33]. Moreover, lymph nodes are connected with blood vessels, enabling the exchange of water, proteins, and cells between the lymph and blood [12, 34]. Numerical studies suggest that approximately 10% of the incoming lymph flow is absorbed directly into the blood vessels within the lymph node [34], while the remainder continues toward the thoracic or right lymphatic ducts.

After entering the systemic circulation, therapeutic proteins are distributed to tissues and eliminated via intracellular catabolism [9, 35]. A critical determinant of bioavailability for Fc-containing proteins (such as mAbs) is the neonatal Fc receptor (FcRn). FcRn protects these proteins from lysosomal degradation through a pH-dependent recycling [9, 36]. FcRn-expressing cells, such as radiosensitive hematopoietic cells, are present at the injection site and within the lymphatic system, where they protect therapeutic proteins from pre-systemic catabolism [37–40]. In murine models, the presence of FcRn has been shown to increase the bioavailability of subcutaneously administered IgG1 by approximately three-fold compared to FcRn-deficient controls [41].

## Factors Affecting Lymphatic Uptake Rate of Large Therapeutic Proteins

### Injection Site

The arm, thigh, and abdomen are primary sites for SC administration. These locations vary in SC layer thickness, capillary and initial lymphatic density, and the anatomical path to systemic circulation. Such variation affects the PK of large therapeutic proteins. Existing studies [8, 42] suggest that smaller proteins or those with a shorter time to maximum concentration ($T_{\max }$) or half-life ($T_{1/2}$) are more susceptible to site-dependent absorption.

Large therapeutic proteins are transported via the lymphatic system, and the uptake rate is sensitive to local lymphatic flow. This flow is governed by generation of interstitial fluid, which depends on blood flow, hydrostatic pressure, and vascular permeability and densities. Studies reported that lymphatic flow in the human thigh is generally higher than that in the abdomen and arm [8, 43, 44]. Furthermore, Body Mass Index (BMI) exhibits an inverse relationship with lymph flow [44], while breast cancer induces significant regional variations [45, 46].

The thickness of the SC layer determines the morphology of the injection plume [47–52]. A thinner SC layer usually results in a flatter, ellipsoidal plume shape [53, 54]. This configuration distributes the therapeutic depot closer to the dermis, where initial lymphatic density is highest, thereby accelerating uptake, as suggested by computational simulations [10]. Conversely, a thicker SC layer allows for a more spherical plume with a deeper reach into the adipose tissue. It is worth noting that the computational models [10, 54] assume initial lymphatics are confined exclusively to the dermis, prescribing both the density and permeability of the capillaries and initial lymphatics. Because the spatial architecture of the capillaries and initial lymphatics significantly affects the convective and diffusive transport of large therapeutic proteins within the SC tissue, high-resolution experimental characterization of these structural parameters is critically needed. Furthermore, current computational models prescribe fixed pressures within the initial lymphatics and capillaries, which determines the interstitial fluid flow rates. To improve model accuracy, experimental measurements of the in vivo convective velocity and protein diffusivity within the SC tissue are essential.

**Fig. 3:**
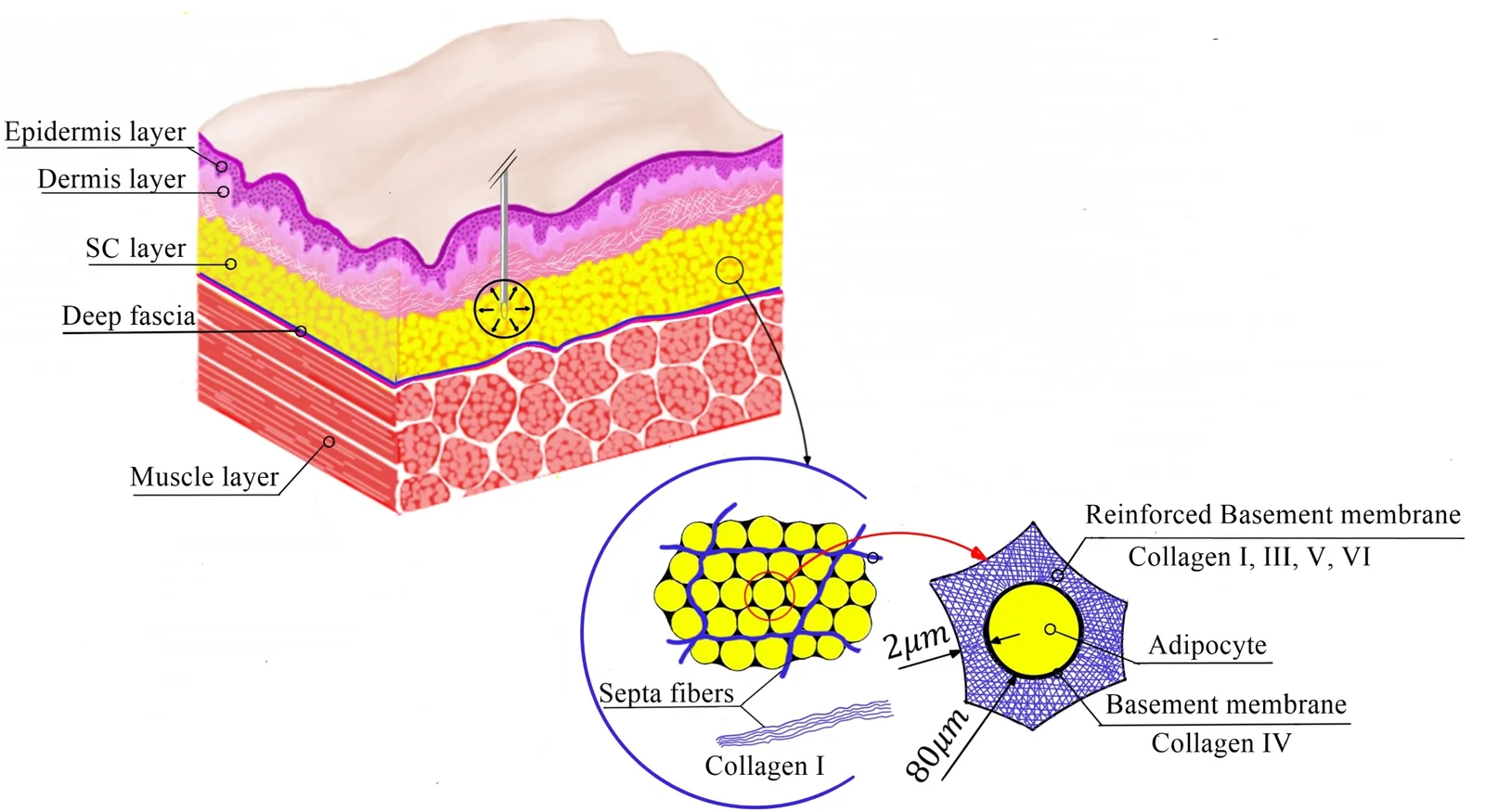
ECM structure of the SC layer. Reproduced with permission from International Journal of Pharmaceutics [58].

The ECM structure of the SC layer (Fig. 3) also contributes to site-dependent differences in plume morphology and lymphatic uptake. The majority of the collagenous part of the ECM in the SC layer is formed by the network of septa fibers [6], and the remaining part includes a reinforced basement membrane around adipocyte cells. Septa fibers are type I collagen fibers aligned together in one direction, while reinforced basement membranes are networks of two-dimensional woven fibers of collagen types I, III, V, and VI [55]. Experiments [56, 57] show preferential drug transport along septa fibers due to their higher permeability relative to the surrounding adipose tissue. Thus, the density and orientation of the septa network affects the morphology of the injection plume, thereby influencing the subsequent lymphatic uptake rate.

**Fig. 4:**
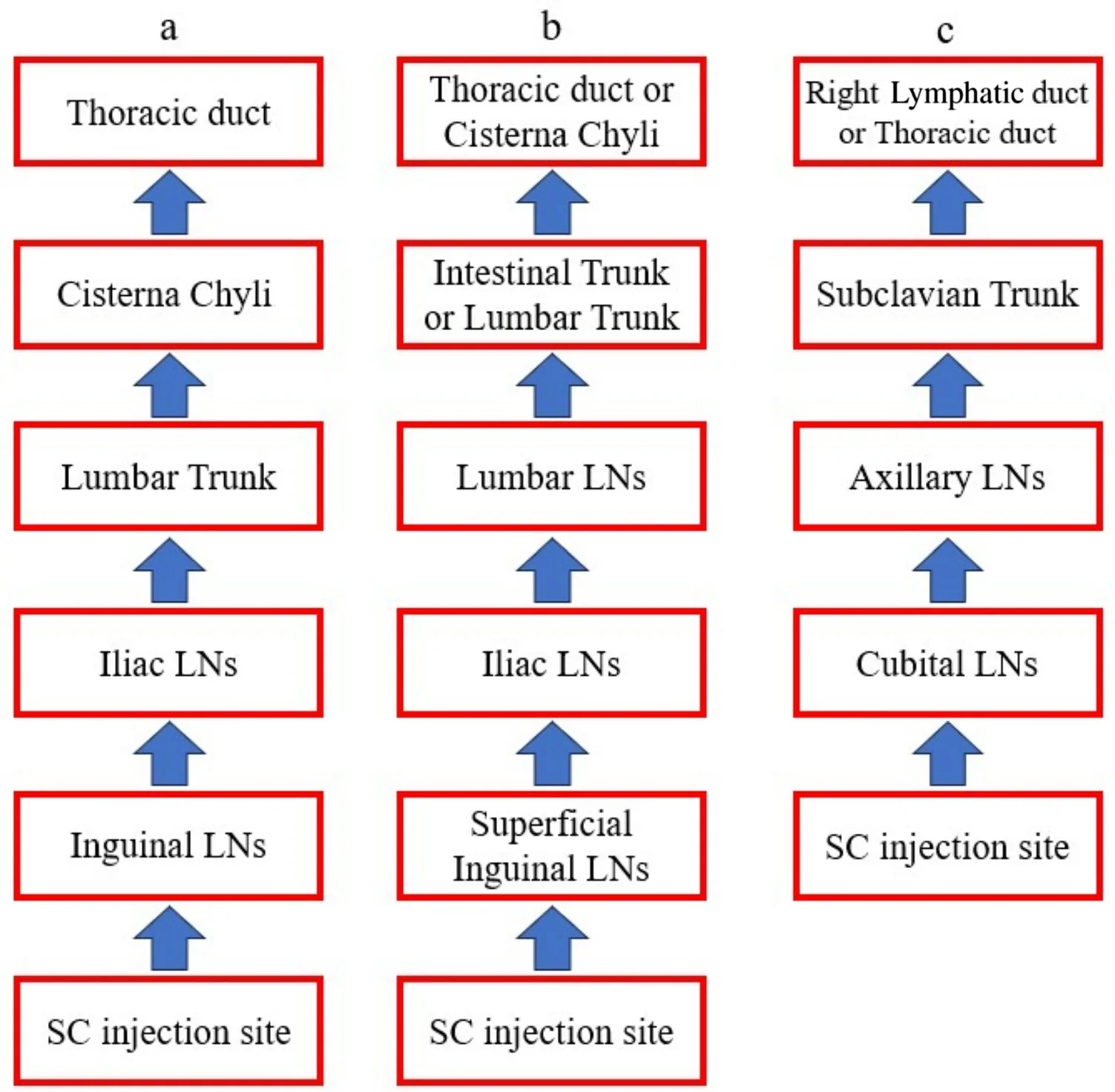
The path to systemic circulation from (**a**) thigh, (**b**) abdomen, and (**c**) arm. LN refers to lymph nodes. Reproduced with permission from Journal of Pharmacy and Pharmaceutical Sciences [59].

Furthermore, the injection location dictates the drainage path to the systemic circulation. Under resting conditions, approximately 120 ml/h of lymph enters the bloodstream: 20 ml/h via the right lymphatic duct (draining the upper right quadrant, including the right arm) and 100 ml/h via the thoracic duct (draining the remainder of the body) (Fig. 1). These distinct pathways subject drug molecules to varying transit distances, travel times, and numbers of lymph nodes (Fig. 4). Consequently, the choice of injection location, such as the arm, abdomen, and thigh, influences the lymphatic uptake of subcutaneously administered large therapeutic proteins [59].

### Delivery Parameters

#### Injection Device

Delivery parameters, including device mechanics and drug formulation, can impact the PK of large therapeutic proteins. Demonstrating PK bioequivalence between the clinical trial device and the commercial device is a key regulatory requirement.

The choice of delivery system depends on characteristics of therapeutic proteins, patient needs, and manufacturing considerations [3, 60, 61]. The two most prevalent devices for the SC administration of mAbs are prefilled syringes (PFS) and AIs. PFS is a manual delivery system often administered by a healthcare professional. To avoid intramuscular injection, the needle is typically inserted at a 45$^{\circ }$ angle into a pinched tissue. Thus, the injection depth, angle, and flow rate vary greatly based on user technique. By contrast, designed for patient self-administration, AIs utilize a standard 90$^{\circ }$ injection angle with controlled needle extension and a mechanical spring to ensure a consistent, often higher, delivery rate compared to PFS.

**Fig. 5:**
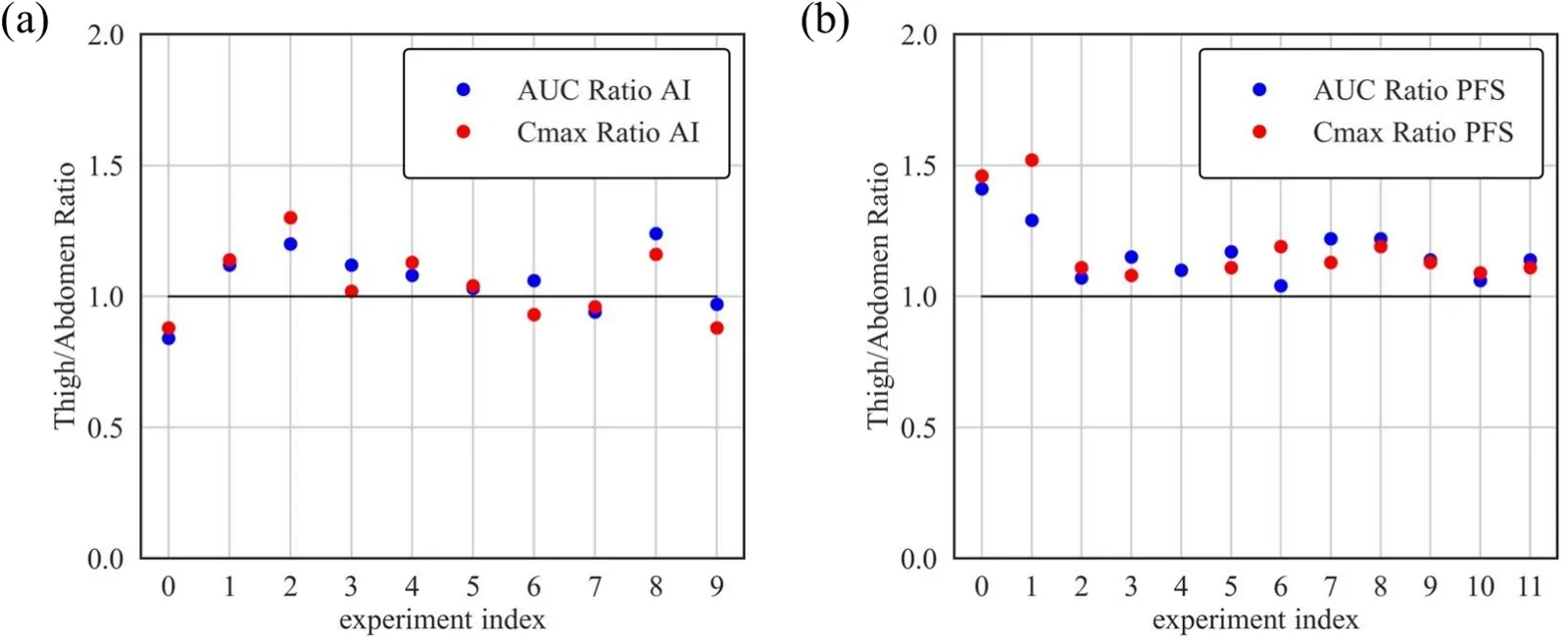
The roles of injection devices ((**a**) autoinjectors (**b**) prefilled syringes) and injection location on the area under the curve (AUC) and $C_{\max }$ of mAbs extracted from [8].

**Fig. 6:**
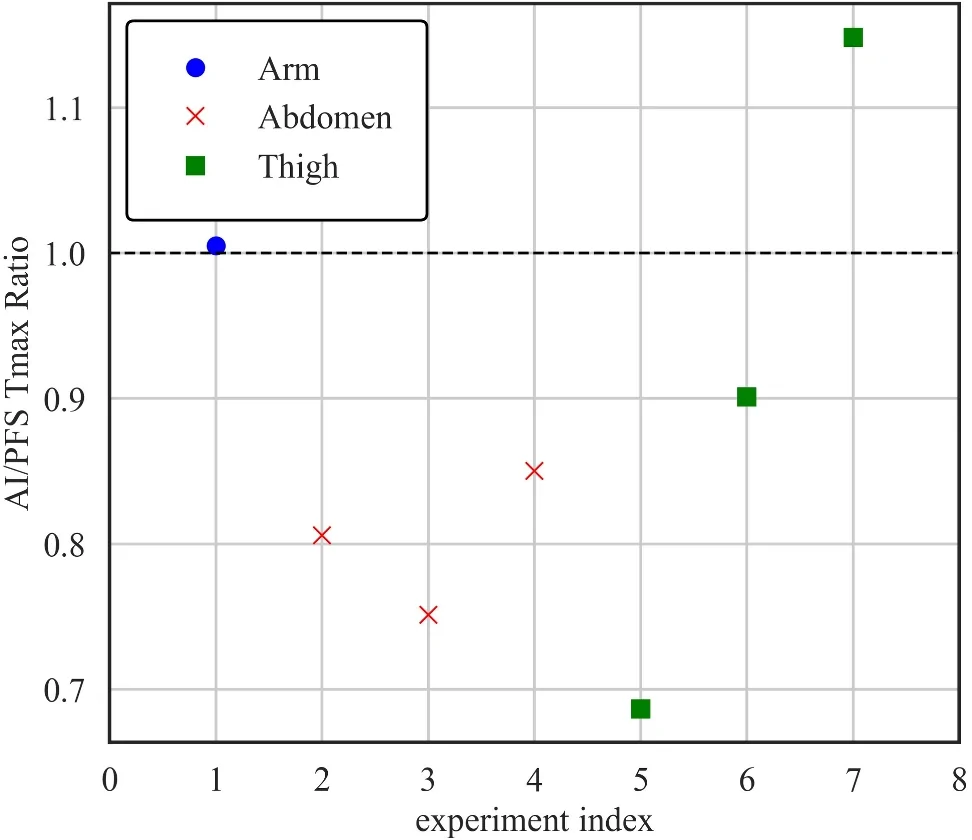
The effect of injection devices on $T_{\max }$ of mAbs based on data from [62–64].

Clinical PK data indicate that injection devices affect the SC absorption of large therapeutic proteins [8]. Figure 5 compares the PK (the area under the curve (AUC) and the maximum serum concentration ($C_{\max }$)) of thigh versus abdomen injections for various mAbs. When using AIs, these ratios are close to 1 and even below 1 in specific instances, whereas PFS administration consistently yields ratios exceeding 1. Furthermore, Figure 6 indicates that $T_{\max }$ (the time to peak plasma concentration) is mostly shorter for AIs than for PFS across multiple datasets. Compartmental modeling investigations [11] have demonstrated that $T_{\max }$ decreases with an increasing rate of lymphatic uptake, assuming a constant local degradation rate and a fixed transit time from the lymphatic system to the systemic circulation. Given that the therapeutic protein and injection location remain identical between devices, the degradation rate and the lymphatic transit rates may be considered to remain unchanged. Under this assumption, the shorter $T_{\max }$ observed with AI-based delivery implies an accelerated initial lymphatic uptake rate. However, this uptake rate encapsulates a transport process governed by therapeutic protein volume, deposition depth, and delivery rate. Consequently, resolving these underlying mechanics requires more sophisticated, physically grounded computational and experimental investigations. These findings underscore the critical role of device mechanics and local tissue biomechanics in modulating the systemic absorption profiles of therapeutic mAbs.

#### Injection Rate and Volume

Injection rate and volume are important parameters that influence the mechanical response of the SC tissue and the resulting morphology of the drug plume. Reported injection rates vary significantly, from $0.33\; \mathrm {ml/min}$ [65, 66] to $120\;\mathrm {ml/min}$ [3]. While higher injection rates create higher interstitial pressure, which theoretically could force open lymphatic intercellular junctions to increase flow [67, 68], many experimental [65, 69] and computational studies [54, 70] suggest that this post-injection pressure decays rapidly, and its effect on long-term drug uptake is minor [58, 70]. However, these computational models assume a poroelastic tissue [54, 58, 70] undergoing infinitesimal elastic deformation, where lymphatic uptake is driven by the pressure gradient between the interstitial space and a fixed baseline pressure prescribed within the initial lymphatics. Additionally, other mechanical phenomena that do not dissipate as rapidly as the local interstitial pressure, such as tissue fracturing, are not captured, potentially leading to an underestimation of the influence of injection rate on overall transport.

A more significant consequence of high injection rates is the alteration of plume morphology. High local pressures can increase tissue permeability by inducing hydraulic fracturing of the interstitial matrix [53, 71]. These micro-cracks create stochastic, high-permeability pathways that unevenly expand the plume. Because initial lymphatics are non-uniformly distributed and concentrated near the dermis, the alteration in plume morphology affects the drug delivery path and subsequent lymphatic uptake profile [10].

Injection volume similarly modulates PK of the SC administration by altering therapeutic protein dispersion patterns [42, 68]. Injection volumes greater than 2 ml encounter significant resistance from the collagenous scaffold and viscous hyaluronic acid (HA) within the SC tissue [67, 72, 73]. Experimental data demonstrate that larger volumes often lead to drug accumulation along the fascia layer, which separates superficial and deep adipose tissue [57]. This distribution may explain why higher injection volumes lead to different lymphatic uptake rates, as initial lymphatics are more concentrated near the dermis. As with injection rate, higher volumes also elevate interstitial pressure, but this effect remains transient and dissipates shortly after administration [54, 65].

#### Injection Depth

The primary consideration for the depth of SC injection is to ensure needle penetration into the SC tissue while avoiding the underlying muscle. While a 4 mm depth is generally sufficient for SC delivery [74], manual techniques with PFS often result in deeper, more variable delivery compared to the standardized depth of AIs.

Regulatory and bridging studies identify injection depth as a potential factor affecting lymphatic uptake and systemic PK [75, 76]. Computational investigations [10, 77] indicate that deeper SC delivery disperses the therapeutic protein into the lower regions of the SC tissue, even though interstitial pressure remains largely independent of injection depth. When using a PFS, clinical protocols typically require pinching or stretching the skin [78] and inserting the needle at an angle of $45^\text {o}$ to $90^\text {o}$. Given that the nominal needle length for PFS is 12.7 mm, the injection depth is approximately 6-10 mm below the unpinched skin surface. In contrast, autoinjectors are held firmly against the skin at an angle of $90^\text {o}$ [78]. Although their nominal needle lengths range from 4 to 12.7 mm, mechanical constraints and device-induced skin compression generally limit the injection depth to approximately 4-8 mm. Thus, the deposition depth of therapeutic proteins for autoinjectors is typically shallower than that of manual PFS. Since initial lymphatics are most abundant near the dermal-SC interface, shallow injections localize the drug plume closer to this boundary, thereby likely facilitating more rapid uptake than deeper administrations [10]. However, these computational models lack experimental validation of their predicted plume dynamics during and post-injection. Experimental studies [57, 79] have employed ex vivo micro-computed tomography to characterize the effects of needle length, injection speed, volume, and viscosity on the spatial deposition of therapeutic proteins in the minipig SC tissue. These quantified spatial profiles provide valuable datasets for validating high-fidelity computational injection simulations.

####  Solution Viscosity

Large-volume SC injection is challenging primarily due to the resistance of the ECM to fluid flow. Consequently, high-dose SC formulations are often highly concentrated to minimize the injection volume. However, this increases solution viscosity and affects the convective velocity and distribution of therapeutic proteins in the tissue [10, 58]. Computational studies [10] suggest that higher viscosity of the therapeutic proteins solution reduces the initial lymphatic uptake rate, as protein molecules traverse the ECM more slowly to reach the dermal lymphatic network.

Concentration-dependent viscosity for mAbs is frequently characterized using models such as the Mooney equation [80–83]:

1 $$\begin{aligned} \eta /\eta _0 =\exp \left( \frac{bC}{1-\frac{C}{C_{\text {p}}}}\right) . \end{aligned}$$

**Fig. 7:**
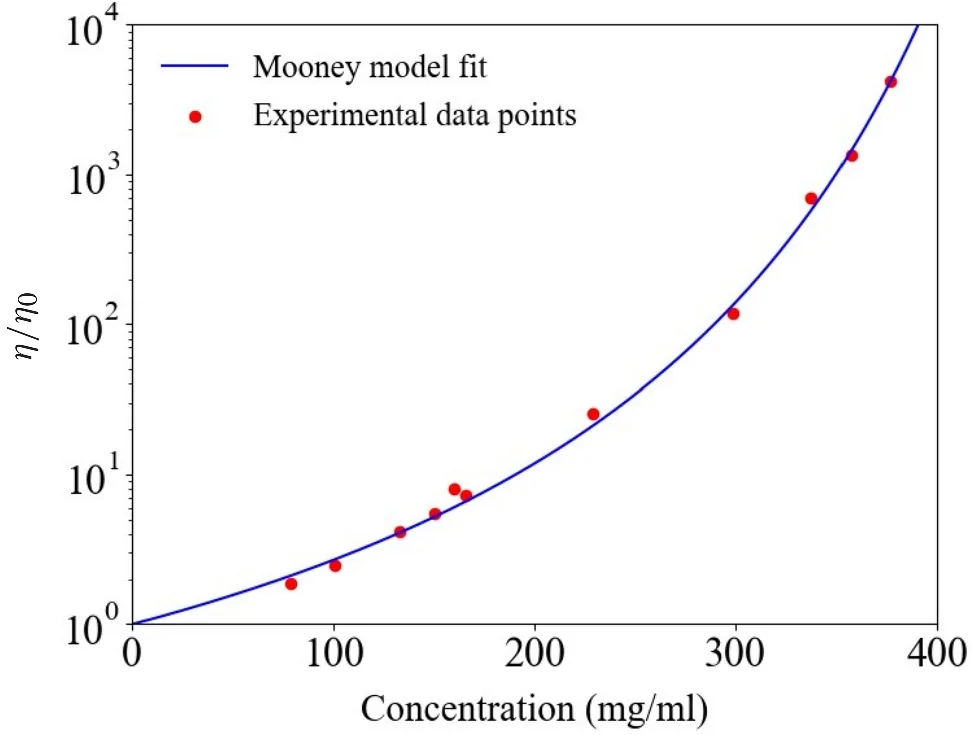
Concentration dependent viscosity of IgG solutions taken from [82] along with Mooney model fit [80, 81, 83].

**Fig. 8:**
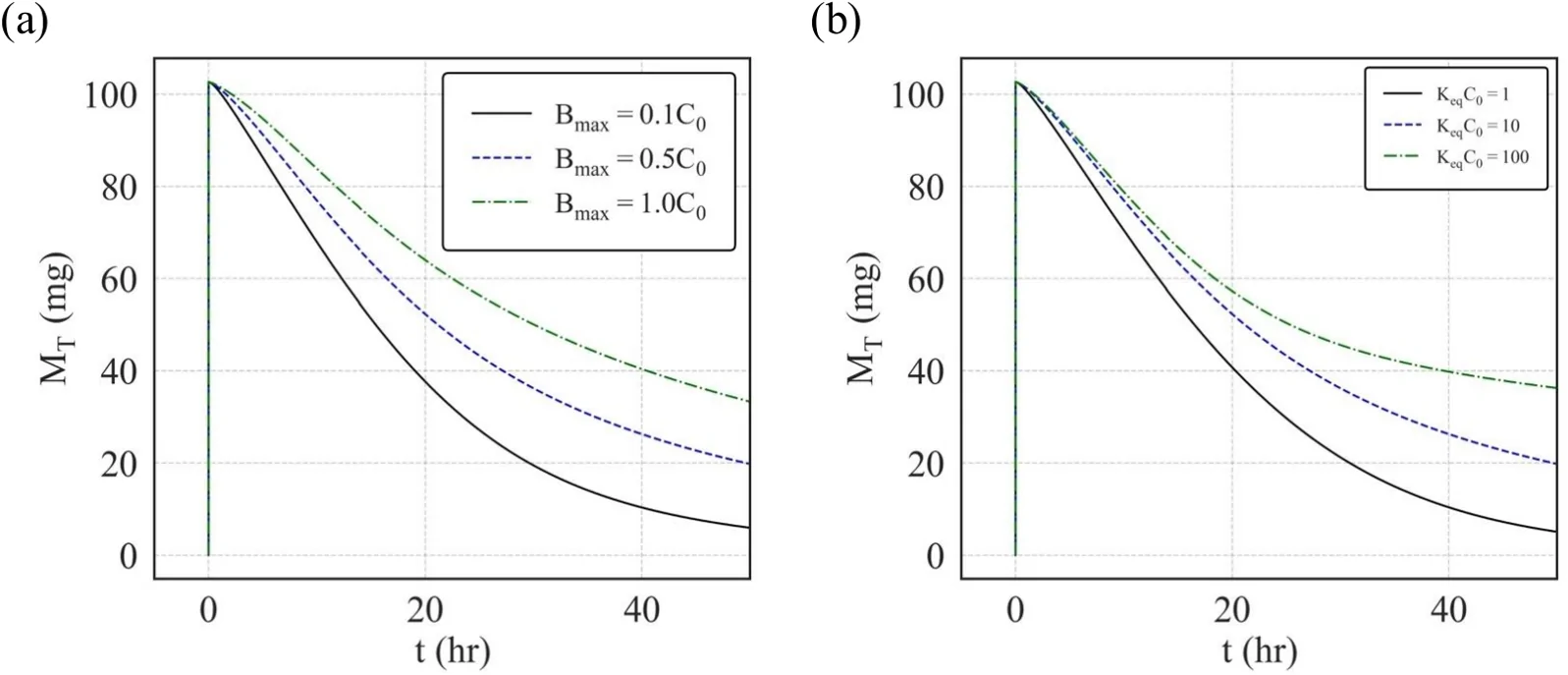
The effect of binding on the mass of mAbs remaining in the tissue ($M_{T}$). Reproduced by permission from Computers in Biology and Medicine [10] for a) different values of $B_{\max }$ and b) different values of $K_{eq}$. Here, $C_0$ is the concentration of therapeutic proteins.

Here, $\eta /\eta _0$ is the relative viscosity, where η and $\eta _0$ are the viscosities of the protein solution and the solvent (e.g., buffer), respectively. Variable *C* represents the protein concentration with the unit of mg/ml, *b* is associated with the intrinsic viscosity, and $C_{\text {p}}$ quantifies crowding effects and steric constraints. For the IgG experimental data shown in Fig. 7, the values of b and $C_{\text {p}}$ are $8.25 \times 10^{-3} \, \text {ml/mg}$ and $600.54 \, \text {mg/ml}$, respectively.

### Molecular Characteristics of Large Therapeutic Proteins

#### Molecular Weight

The MW of large therapeutic proteins is a critical determinant of their absorption, distribution and elimination. Large molecules ($> 40 \; \textrm{kDa}$) rely almost exclusively on lymphatic uptake as their primary route to the systemic circulation [6]. They also experience hindered diffusion in the ECM due to steric hindrance and mechanical barrier. Furthermore, MW may restrict passage through the primary valves of initial lymphatics, particularly when tissue deformation is insufficient to fully open these endothelial junctions.

#### Charge and Binding

Molecular charge significantly influences the lymphatic uptake of large therapeutic proteins [84]. Since most antibodies possess isoelectric points exceeding the physiological pH of SC tissue, they carry a net positive charge upon injection. This promotes electrostatic binding to negatively charged components of the ECM, thereby hindering interstitial transport and delaying entry into initial lymphatic vessels [17, 68, 85]. Furthermore, these highly charged molecules tend to aggregate, compromising both stability and transport dynamics [86]. Moreover, molecular charge impacts solution viscosity, which directly modulates the initial spreading and distribution of the bolus during and after SC injection [87, 88].

Recent quantitative models have characterized the binding of mAbs to SC tissue and its subsequent impact on reducing lymphatic uptake rates [10, 89]. These studies utilize the following Langmuir isotherm to estimate the amount of bound proteins:

2 $$\begin{aligned} C_B = \frac{C\cdot K_{eq}}{1+ C\cdot K_{eq}}B_{\max }, \end{aligned}$$

where $C_B$ is the bound protein concentration, $K_{eq}$ is the adsorption equilibrium constant, and $B_{\max }$ is the maximum binding capacity of the tissue. As illustrated in Fig. 8, the temporal profile of the total protein mass remaining at the injection site is dependent on the binding parameters. In these models, higher capacity ($B_{\max }$) or affinity ($K_{eq}$) leads to prolonged retention of proteins in the tissue, effectively reducing the rate of lymphatic uptake. Note that multiple binding models are available to describe this adsorptive interaction [90, 91]. Here, the Langmuir isotherm is adopted as a tractable first approximation, assuming reversible and saturable retention of mAbs in the SC tissue. Although sensitivity analyses highlight the effect of binding kinetics on absorption and transport, the isotherm parameters ($B_{\max }$ and $K_{eq}$) remain difficult to measure independently, and a direct experimental binding isotherm for the mAb–ECM system is still lacking.

**Fig. 9:**
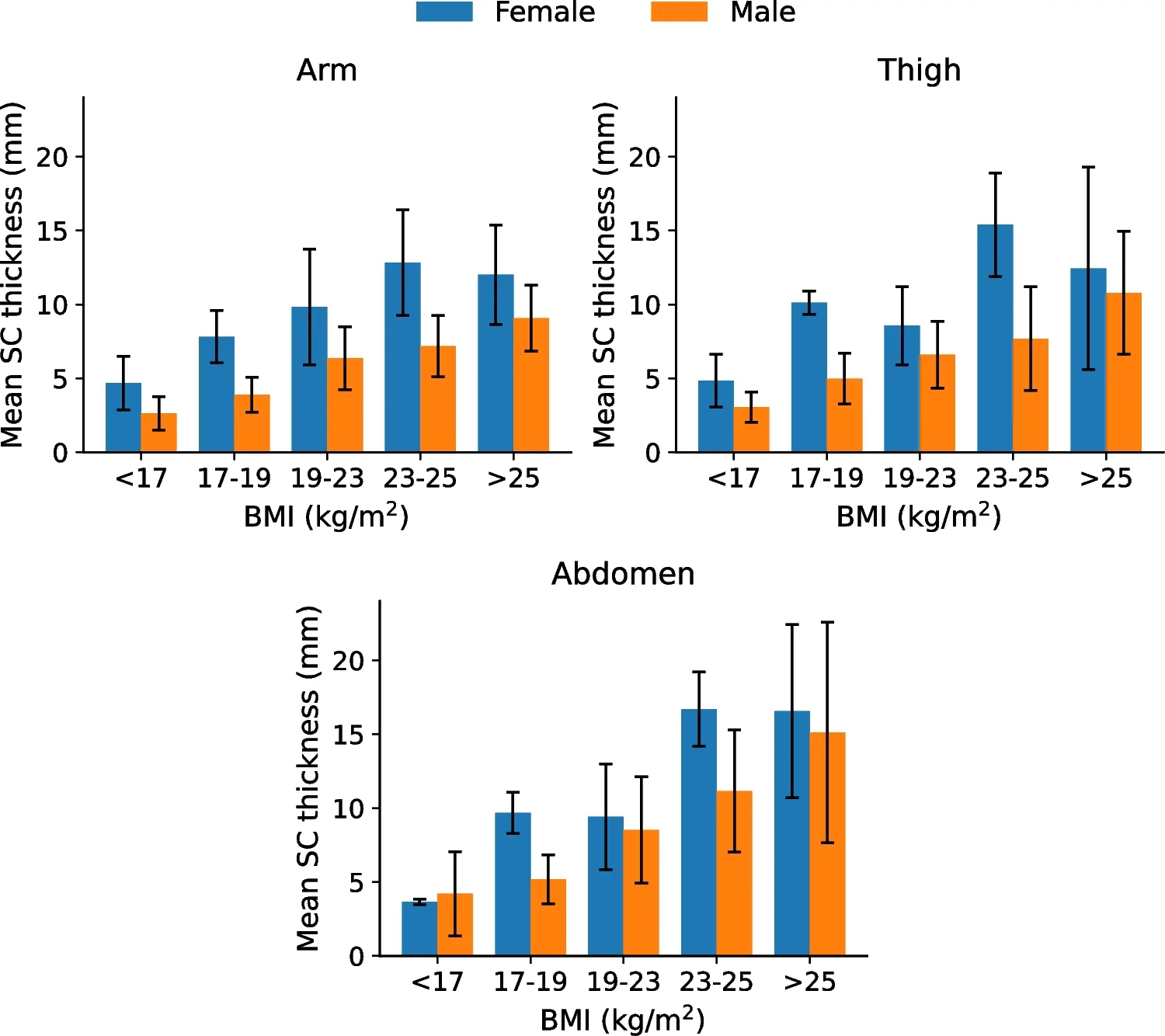
Mean subcutaneous layer thickness across different anatomical locations and BMI ranges. Data are adapted from [104].

#### Coformulation with Hyaluronidase

Historically, high-volume SC injections were considered limited by perceived patient discomfort [59], but this assumption has been challenged by recent clinical evidence [92]. To enhance tolerability and accommodate larger volumes, various strategies have been explored, most notably the co-formulation with recombinant human hyaluronidase (rHuPH20) [69, 72, 93]. Hyaluronidase is an enzyme that degrades the HA, a primary structural component of the SC ECM. This enzymatic digestion is non-invasive and reversible, as HA levels typically restore within 24 to 48 hours [6]. Mechanistically, HA degradation increases interstitial permeability, resulting in more rapid drug dispersion and a significant reduction in infusion-induced interstitial pressure [69, 74]. Furthermore, experimental studies in rats [94] observed that co-administration with rHuPH20 increased the SC bioavailability of cetuximab from 67% to 80% and elevated its lymphatic recovery from 35.8% to 49.4%; in contrast, rHuPH20 co-administration did not alter plasma exposure for trastuzumab. In addition, a clinical study [95] evaluating SC administration of crenezumab in 68 healthy participants (aged 50-80 years) observed that crenezumab exhibited approximately dose-proportional PK, with an SC bioavailability of 66% that remained independent of rHuPH20 co-administration.

### Physiological Conditions and Extrinsic Factors

The impact of physiological factors, such as age, gender, and BMI, on lymphatic uptake rates remains a critical area of investigation. In particular, BMI has been shown to inversely correlate with lymphatic flow rates [44]. Note that BMI affects multiple interrelated anatomical and mechanical properties that may affect uptake. For example, the SC layer thickness generally increases with BMI (Fig. 9), and at a given BMI, females display a higher SC layer thickness than males [47]. In addition, the density and orientation of the septa fibers and fascia layers vary with age and BMI [6, 96, 97], while SC thickness tends to decrease with age [6]. These structural differences can alter the morphology of the injection plume. Overall, further experimental studies are needed to disentangle the individual contributions of gender, age, and BMI.

Beyond these intrinsic physiological factors, extrinsic stimuli can also modulate lymphatic flow and thereby influence uptake. The lymphatic circulation is primarily driven by the periodic oscillation of collecting lymphatics, which involve both intrinsic and extrinsic mechanisms. Extrinsic factors, such as the pulsation of surrounding blood vessels, exercise, and external massage, cause periodic deformation of the lymphangion, significantly influencing lymph flow [12, 98, 99]. Under the influence of external factors such as exercise and massage, the lymph flow rate increases [100–103], suggesting a higher lymphatic uptake rate from initial lymphatics, potentially leading to faster drug delivery into the systemic circulation.

## Experimental and Computational Studies of Lymphatic Uptake Rate

Quantifying the rate of lymphatic uptake is critical for evaluating the efficacy of SC drug delivery. Two primary experimental frameworks have been conducted to assess the rate of lymphatic uptake. The first approach measures the velocity of lymph flow within collecting vessels, typically utilizing lymphoscintigraphy to measure the rate of fluid transport from the vicinity of a tumor to the sentinel lymph node, where tumor spread potentially begins [43, 44]. This technique involves the intradermal injection of radiolabeled tracers, followed by dynamic imaging to track their transport through the lymphatic network [43, 105, 106]. The flow rates are location-dependent (Table 1, adapted from [43]), and experimental studies [44] have further explored the effects of age, gender, and body mass index on lymphatic flow rates.

**Table 1:** Lymphatic flow velocity reproduced with permission from [43].

Location	Average lym. flow velocity (cm/min)
Head and neck	1.5
Anterior trunk	2.8
Posterior trunk	3.9
Arm and shoulder	2.0
Forearm and hand	5.5
Thigh	4.2
Leg and foot	10.2

**Fig. 10:**
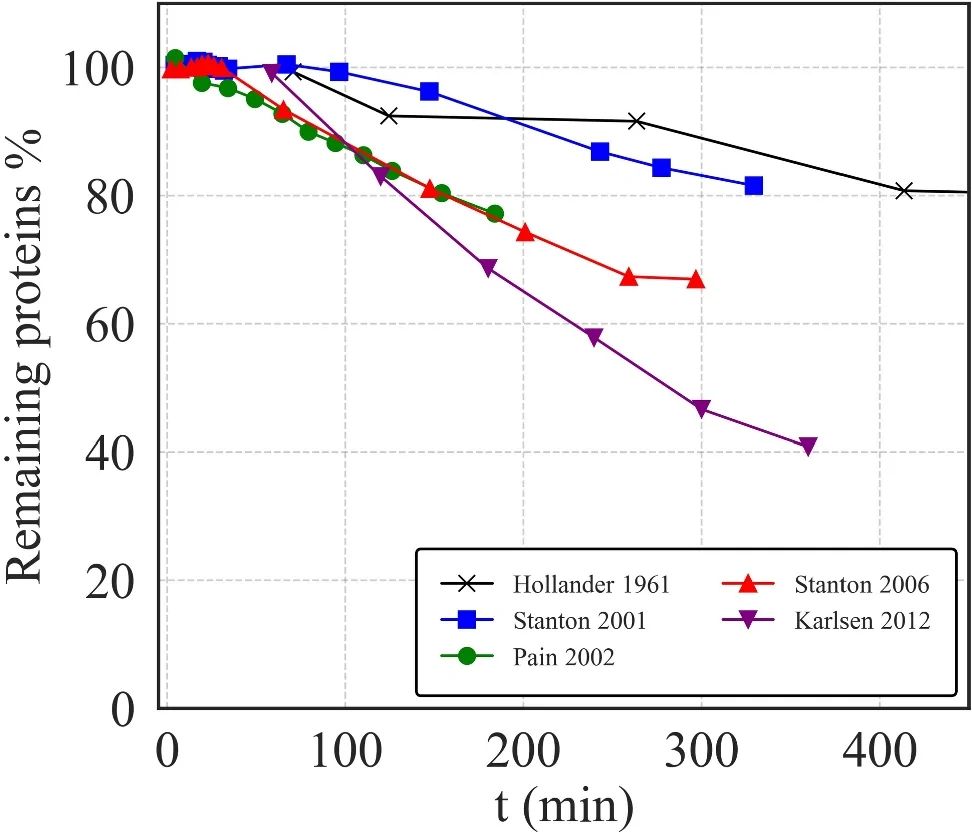
Percentage of remaining proteins in the tissue after SC injection [45, 46, 108–110].

The second approach, as detailed in [45, 46, 107–110], utilizes the depot clearance test to evaluate lymphatic absorption following the SC injection. This methodology involves labeling a protein with a tracer (typically a radionuclide) and monitoring the residual radiolabeled proteins at the injection depot over several hours (Fig. 10). The decay in protein mass remaining at the site is modeled using first-order kinetics:

3 $$\begin{aligned} \frac{dM_T}{dt} = -k_{abs}M_T \end{aligned}$$

where $M_T$ is the total mass of injected proteins remaining at the injection site, and $k_{abs}$ is the lymphatic uptake rate constant. A comprehensive summary of these experimental studies and their associated parameters is provided in Table 2.

**Table 2:** Depot clearance experiments.

Proteins	Tracers	Volumes	Inj. locations	Ref.
HSA	${}^{131}\textrm{I}$	0.03 and 0.05 ml	Forearm and leg	[108]
IgG	${}^{99m}\textrm{Tc}$	0.2 ml	Forearm	[45]
HSA, HIgG	${}^{99m}\textrm{Tc}, {}^{111}\textrm{In}$	0.2 ml	Second dorsal web space of hand	[109]
IgG	${}^{99m}\textrm{Tc}$	0.2 ml	Forearm	[46]
BSA	Fluorescent	0.5 and 1 μl	Mouse hind paw	[110]

Figure 10 shows an initial lag phase during which the protein level at the injection site remains at 100%. Notably, most existing literature and pharmacokinetic models overlook this delay, assuming immediate first-order kinetics. As discussed in [10], this phase is attributed to the time required for the biotherapeutic molecules to reach the initial lymphatic capillaries in the dermis layer. This transport duration is sensitive to several factors previously addressed in this review, including injection depth, molecule-tissue binding affinity, and solution viscosity [54].

The experimental frameworks described above have provided the necessary validation data for several recently developed computational models [10, 54, 111]. These works employ a variety of constitutive tissue models, ranging from rigid porous media [112, 113] and linear poro-elasticity [54, 58, 77, 111] to more complex nonlinear poro-hyperelastic [114–116] and poro-viscoelastic models [117], to characterize the interstitial pressure buildup, drug distribution, and subsequent transport toward the initial lymphatics. Regarding the lymphatic interface, researchers typically adopt the following two approaches: explicitly modeling the geometry of lymphatic vessels [89, 113, 118, 119] or incorporating lymphatics as distributed sink terms within the fluid continuity equations [58, 120].

Recent sensitivity analyses have highlighted how tissue biomechanical properties affect the depot formation and drug distribution [58]. The impact of BMI on depot shape is also quantified in [120]. Additionally, literature [10] conducted a comprehensive study on how the injection device, injection location, and the characteristics of mAbs affect the lymphatic uptake rate. Their findings indicate that the binding affinity of mAbs to the SC tissue is a critical factor in affecting pre-systemic absorption kinetics.

## Limitations and Knowledge Gaps

Despite significant computational and experimental progress in characterizing the SC administration of large therapeutic proteins, the mechanisms governing drug absorption remain elusive, and quantitative studies are still needed. Addressing these gaps is critical for optimizing therapy and improving the translation of preclinical findings to human applications.

First, the mechanisms underlying pre-systemic clearance require further elucidation. In particular, kinetic parameters associated with enzymatic degradation within SC tissues or during lymphatic transport, uptake by local immune cells (e.g., macrophages and dendritic cells), and binding to ECM components need to be quantified. Such information is valuable for developing high-fidelity computational models. Additionally, the influence of therapeutic-protein molecular characteristics on lymphatic uptake rates needs further investigation. The use of large therapeutic proteins, particularly mAbs, to treat chronic diseases continues to grow, with an increasing number of newly identified candidates. These proteins vary in molecular weight, charge, and hydrophobicity/lipophilicity. Such differences likely contribute to variability in the transport and binding of molecules within SC tissues, lymphatic uptake, and transport and degradation within lymphatic vessels. However, quantitative models linking bioavailability to the characteristics of therapeutic proteins have yet to be established.

Second, the effect of injection devices and delivery parameters on lymphatic uptake rates remains unclear. For instance, AIs and PFS can yield different PK profiles, making it difficult to bridge different devices during therapeutic-protein development. Additionally, while transient pressure buildup appears to have a negligible effect on lymphatic uptake, injection rate and depth can significantly affect the initial morphology of the drug depot and its proximity to dermal lymphatic capillaries. A robust, experimentally validated framework to quantify these device-specific effects is currently lacking.

Third, experimental studies suggest that physiological factors, such as BMI, age, and gender, can influence lymphatic uptake rates and the bioavailability of large therapeutic proteins. However, these findings are largely qualitative and require further investigation to enable more comprehensive, quantitative models.

## Conclusions

Optimizing the therapeutic performance of large proteins delivered subcutaneously requires a clear understanding of the factors that govern lymphatic uptake. In this review, we synthesized current evidence on injection location, physiological transport conditions, and protein attributes. We also highlighted the impact of device- and product-related delivery parameters on lymphatic uptake, which is critical in drug development where multiple devices or formulations must be bridged to demonstrate bioequivalence. These insights provide a foundation for the rational design of next-generation protein therapies that achieve improved biodistribution and efficacy while enabling patient-friendly SC administration.

Future progress will require mechanistic, quantitative studies that integrate biology, formulation, and device-related effects. Developing experimentally validated predictive models that link lymphatic uptake to systemic exposure across products and diverse patient populations will also be valuable. Specifically, advances in biomaterials, microfluidic technologies, and real-time monitoring offer exciting prospects for optimizing SC injections. Development in these fields will enable personalized injection strategies informed by patient-specific anatomical and physiological parameters. Enhanced diagnostic tools and real-time monitoring can become integral to optimizing drug utilization and minimizing variability in therapeutic outcomes. As mechanistic insights deepen, standard procedures for the administration of large therapeutic proteins can evolve to include adaptive delivery systems that adjust to patient physiology.

## References

[1] Dimitrov DS. Therapeutic proteins. Therapeutic Proteins: Methods and Protocols. 2012;1–26.

[2] Reilly RM, Domingo R, Sandhu J. Oral delivery of antibodies: future pharmacokinetic trends. Clin Pharmacokinet. 1997;32:313–23. 9113439 10.2165/00003088-199732040-00004

[3] Zhong X, Guo T, Vlachos P, Veilleux J-C, Shi GH, Collins DS, et al. An experimentally validated dynamic model for spring-driven autoinjectors. Int J Pharm. 2021;594:120008. 33189808 10.1016/j.ijpharm.2020.120008

[4] Sree V, Zhong X, Bilionis I, Ardekani A, Tepole AB. Optimizing autoinjector devices using physics-based simulations and Gaussian processes. J Mech Behav Biomed Mater. 2023;140:105695. 10.1016/j.jmbbm.2023.105695 36739826

[5] Li Z, Du X, Huang SM, Wang Y-MC. Pharmacokinetics-bridging between autoinjectors and prefilled syringes for subcutaneous injection: Case examples revealing a knowledge gap. Clin Pharmacol Therapeutics. 2024;115(3):404–7. 10.1002/cpt.3145 38167787

[6] Richter WF, Bhansali SG, Morris ME. Mechanistic determinants of biotherapeutics absorption following SC administration. AAPS J. 2012;14:559–70. 22619041 10.1208/s12248-012-9367-0 PMC3385825

[7] Viola M, Sequeira J, Seiça R, Veiga F, Serra J, Santos AC, et al. Subcutaneous delivery of monoclonal antibodies: How do we get there? J Control Release. 2018;286:301–14. 30077735 10.1016/j.jconrel.2018.08.001

[8] Zou P, Wang F, Wang J, Lu Y, Tran D, Seo SK. Impact of injection sites on clinical pharmacokinetics of subcutaneously administered peptides and proteins. J Control Release. 2021;336:310–21. 34186147 10.1016/j.jconrel.2021.06.038

[9] Ryman JT, Meibohm B. Pharmacokinetics of monoclonal antibodies. CPT Pharmacomet Syst Pharmacol. 2017;6(9):576–88. 10.1002/psp4.12224 PMC5613179 28653357

[10] Rahimi E, Li C, Zhong X, Shi G, Ardekani A. The role of initial lymphatics in the absorption of monoclonal antibodies after subcutaneous injection. Computers in Biology and Medicine. 2024; 183:109193. 10.1016/j.compbiomed.2024.109193 39423704

[11] Zhong X, Liu Y, Ardekani AM. A compartment model for subcutaneous injection of monoclonal antibodies. Int J Pharm. 2024;650:123687. 38103705 10.1016/j.ijpharm.2023.123687

[12] Moore JE Jr, Bertram CD. Lymphatic system flows. Annu Rev Fluid Mech. 2018;50:459–82. 29713107 10.1146/annurev-fluid-122316-045259 PMC5922450

[13] Swartz MA. The physiology of the lymphatic system. Adv Drug Deliv Rev. 2001;50(1–2):3–20. 11489331 10.1016/s0169-409x(01)00150-8

[14] Gradel AKJ, Porsgaard T, Lykkesfeldt J, Seested T, Gram-Nielsen S, Kristensen NR, et al. Factors affecting the absorption of subcutaneously administered insulin: effect on variability. J Diabetes Res. 2018;2018(1):1205121. 30116732 10.1155/2018/1205121 PMC6079517

[15] Richter WF, Jacobsen B. Subcutaneous absorption of biotherapeutics: knowns and unknowns. Drug Metab Dispos. 2014;42(11):1881–9. 25100673 10.1124/dmd.114.059238

[16] Swartz MA, Fleury ME. Interstitial flow and its effects in soft tissues. Annu Rev Biomed Eng. 2007;9:229–56. 17459001 10.1146/annurev.bioeng.9.060906.151850

[17] Datta-Mannan A, Estwick S, Zhou C, Choi H, Douglass NE, Witcher DR, Lu J, Beidler C, Millican R. Influence of physiochemical properties on the subcutaneous absorption and bioavailability of monoclonal antibodies. In: MAbs, Taylor & Francis. 2020. vol 12, p. 1770028. 10.1080/19420862.2020.1770028 PMC7531508 32486889

[18] Suami H, Pan W-R, Mann GB, Taylor GI. The lymphatic anatomy of the breast and its implications for sentinel lymph node biopsy: a human cadaver study. Ann Surg Oncol. 2008;15:863–71. 18043970 10.1245/s10434-007-9709-9 PMC2234450

[19] Kolarsick PA, Kolarsick MA, Goodwin C. Anatomy and physiology of the skin. J Dermatol Nurses’ Assoc. 2011;3(4):203–13.

[20] Redondo PdAG, Gubert F, Zaverucha-doValle C, Dutra TPP, Ayres-Silva JdP, Fernandes N, et al. Lymphatic vessels in human adipose tissue. Cell Tissue Res. 2020;379:511–20. 10.1007/s00441-019-03108-5 31776824

[21] Scallan J, Huxley VH, Korthuis RJ. Capillary fluid exchange: regulation, functions, and pathology. In Colloquium Lectures on Integrated Systems Physiology From Molecules to Function. 2010. vol. 2, p. 1–94. Morgan & Claypool Publishers. 21452435

[22] Breslin JW, Yang Y, Scallan JP, Sweat RS, Adderley SP, Murfee WL. Lymphatic vessel network structure and physiology. Compr Physiol. 2018;9(1):207. 30549020 10.1002/cphy.c180015 PMC6459625

[23] Suami H, Scaglioni MF. Lymphedema management: Anatomy of the lymphatic system and the lymphosome concept with reference to lymphedema. In Seminars in plastic surgery. 2018. vol. 32, p. 5, Thieme Medical Publishers. 10.1055/s-0038-1635118 PMC5891651 29636647

[24] Stacker SA, Williams SP, Karnezis T, Shayan R, Fox SB, Achen MG. Lymphangiogenesis and lymphatic vessel remodelling in cancer. Nat Rev Cancer. 2014;14(3):159–72. 24561443 10.1038/nrc3677

[25] Zhang F, Zarkada G, Yi S, Eichmann A. Lymphatic endothelial cell junctions: molecular regulation in physiology and diseases. Front Physiol. 2020;11:509. 32547411 10.3389/fphys.2020.00509 PMC7274196

[26] Elhay S, Casley-Smith J. Mathematical model of the initial lymphatics. Microvasc Res. 1976;12(2):121–40. 979664 10.1016/0026-2862(76)90013-3

[27] Reddy N, Patel K. A mathematical model of flow through the terminal lymphatics. Med Eng Phys. 1995;17(2):134–40. 7735643 10.1016/1350-4533(95)91885-k

[28] Mendoza E, Schmid-Schönbein GW. A model for mechanics of primary lymphatic valves. J Biomech Eng. 2003;125(3):407–14. 12929246 10.1115/1.1568128

[29] Heppell C, Roose T, Richardson G. A model for interstitial drainage through a sliding lymphatic valve. Bull Math Biol. 2015;77:1101–31. 25911590 10.1007/s11538-015-0078-4

[30] Schmid-Schönbein GW. Mechanisms causing initial lymphatics to expand and compress to promote lymph flow. Arch Histol Cytol. 1990;53:107–14. 2252623 10.1679/aohc.53.suppl_107

[31] Hu Z, Zhao X, Wu Z, Qu B, Yuan M, Xing Y, et al. Lymphatic vessel: origin, heterogeneity, biological functions, and therapeutic targets. Signal Transduct Target Ther. 2024;9(1):9. 38172098 10.1038/s41392-023-01723-x PMC10764842

[32] Willard-Mack CL. Normal structure, function, and histology of lymph nodes. Toxicol Pathol. 2006;34(5):409–24. 17067937 10.1080/01926230600867727

[33] Ikomi F, Kawai Y, Ohhashi T. Recent advance in lymph dynamic analysis in lymphatics and lymph nodes. Ann Vasc Dis. 2012;5(3):258–68. 23555523 10.3400/avd.ra.12.00046 PMC3595856

[34] Jafarnejad M, Woodruff MC, Zawieja DC, Carroll MC, Moore J Jr. Modeling lymph flow and fluid exchange with blood vessels in lymph nodes. Lymphat Res Biol. 2015;13(4):234–47. 26683026 10.1089/lrb.2015.0028 PMC4685511

[35] Shah DK, Betts AM. Towards a platform PBPK model to characterize the plasma and tissue disposition of monoclonal antibodies in preclinical species and human. J Pharmacokinet Pharmacodyn. 2012;39(1):67–86. 22143261 10.1007/s10928-011-9232-2

[36] Pyzik M, Kozicky LK, Gandhi AK, Blumberg RS. The therapeutic age of the neonatal Fc receptor. Nat Rev Immunol. 2023;23:415–32. 36726033 10.1038/s41577-022-00821-1 PMC9891766

[37] Ghetie V, Hubbard JG, Kim J-K, Tsen M-F, Lee Y, Ward ES. Abnormally short serum half-lives of IgG in 2-microglobulin-deficient mice. Eur J Immunol. 1996;26(3):690–6. 8605939 10.1002/eji.1830260327

[38] Israel E, Wilsker D, Hayes K, Schoenfeld D, Simister N. Increased clearance of IgG in mice that lack 2-microglobulin: possible protective role of FcRn. Immunology. 1996;89(4):573–8. 9014824 10.1046/j.1365-2567.1996.d01-775.x PMC1456584

[39] Latvala S, Jacobsen B, Otteneder MB, Herrmann A, Kronenberg S. Distribution of FcRn across species and tissues. J Histochem Cytochem. 2017;65(6):321–33. 28402755 10.1369/0022155417705095 PMC5625855

[40] Richter WF, Christianson GJ, Frances N, Grimm HP, Proetzel G, Roopenian DC. Hematopoietic cells as site of first-pass catabolism after subcutaneous dosing and contributors to systemic clearance of a monoclonal antibody in mice. MAbs. 2018;10(5):803–13. 29621428 10.1080/19420862.2018.1458808 PMC6150636

[41] Wang W, Wang E, Balthasar J. Monoclonal antibody pharmacokinetics and pharmacodynamics. Clin Pharmacol Therapeutics. 2008;84(5):548–58. 10.1038/clpt.2008.170 18784655

[42] Kagan L, Turner MR, Balu-Iyer SV, Mager DE. Subcutaneous absorption of monoclonal antibodies: role of dose, site of injection, and injection volume on rituximab pharmacokinetics in rats. Pharm Res. 2012;29:490–9. 21887597 10.1007/s11095-011-0578-3

[43] Uren RF, Howman-Giles R, Thompson JF. Patterns of lymphatic drainage from the skin in patients with melanoma. J Nucl Med. 2003;44(4):570–82. 12679402

[44] Fujiwara M, Sawada M, Kasuya A, Matsushita Y, Yamada M, Fukamizu H, et al. Measurement of cutaneous lymphatic flow rates in patients with skin cancer: area extraction method. J Dermatol. 2014;41(6):498–504. 24909211 10.1111/1346-8138.12506

[45] Stanton A, Svensson W, Mellor R, Peters A, Levick J, Mortimer P. Differences in lymph drainage between swollen and non-swollen regions in arms with breast-cancer-related lymphoedema. Clin Sci. 2001;101(2):131–40. 11473486

[46] Stanton AW, Modi S, Mellor RH, Peters AM, Svensson WE, Levick JR, et al. A quantitative lymphoscintigraphic evaluation of lymphatic function in the swollen hands of women with lymphoedema following breast cancer treatment. Clin Sci. 2006;110(5):553–61. 10.1042/CS20050277 16343054

[47] Gibney MA, Arce CH, Byron KJ, Hirsch LJ. Skin and subcutaneous adipose layer thickness in adults with diabetes at sites used for insulin injections: implications for needle length recommendations. Curr Med Res Opin. 2010;26(6):1519–30. 20429833 10.1185/03007995.2010.481203

[48] Hirsch L, Byron K, Gibney M. Intramuscular risk at insulin injection sites—measurement of the distance from skin to muscle and rationale for shorter-length needles for subcutaneous insulin therapy. Diabetes Technol Therapeutics. 2014;16(12):867–73. 10.1089/dia.2014.0111 25329935

[49] Dou Z, Eshraghi J, Guo T, Veilleux J-C, Duffy KH, Shi GH, et al. Performance characterization of spring actuated autoinjector devices for Emgality and Aimovig. Curr Med Res Opin. 2020;36(8):1343–54. 10.1080/03007995.2020.1783219 32544355

[50] Chan V, Colville J, Persaud T, Buckley O, Hamilton S, Torreggiani W. Intramuscular injections into the buttocks: are they truly intramuscular? Eur J Radiol. 2006;58(3):480–4. 16495027 10.1016/j.ejrad.2006.01.008

[51] Hida T, Ando K, Kobayashi K, Ito K, Tsushima M, Kobayakawa T, et al. Ultrasound measurement of thigh muscle thickness for assessment of sarcopenia. Nagoya J Med Sci. 2018;80(4):519. 30587866 10.18999/nagjms.80.4.519 PMC6295433

[52] Oltulu P, Ince B, Kokbudak N, Findik S, Kilinc F. Measurement of epidermis, dermis, and total skin thicknesses from six different body regions with a new ethical histometric technique. Turkish J Plastic Surg. 2018;26(2):56–61.

[53] Kim H, Park H, Lee SJ. Effective method for drug injection into subcutaneous tissue. Sci Rep. 2017;7(1):1–11. 28852051 10.1038/s41598-017-10110-w PMC5575294

[54] Rahimi E, Aramideh S, Han D, Gomez H, Ardekani AM. Transport and lymphatic uptake of monoclonal antibodies after subcutaneous injection. Microvasc Res. 2022;139:104228. 34547346 10.1016/j.mvr.2021.104228

[55] Comley K, Fleck NA. A micromechanical model for the Young’s modulus of adipose tissue. Int J Solids Struct. 2010;47(21):2982–90.

[56] Thomsen M, Hernandez-Garcia A, Mathiesen J, Poulsen M, Sørensen DN, Tarnow L, et al. Model study of the pressure build-up during subcutaneous injection. PLoS ONE. 2014;9(8):e104054. 25122138 10.1371/journal.pone.0104054 PMC4133188

[57] Thomsen M, Rasmussen CH, Refsgaard HH, Pedersen K-M, Kirk RK, Poulsen M, et al. Spatial distribution of soluble insulin in pig subcutaneous tissue: effect of needle length, injection speed and injected volume. Eur J Pharm Sci. 2015;79:96–101. 26341408 10.1016/j.ejps.2015.08.012

[58] Rahimi E, Gomez H, Ardekani AM. Transport and distribution of biotherapeutics in different tissue layers after subcutaneous injection. International Journal of Pharmaceutics. 2022;626:122125. 10.1016/j.ijpharm.2022.122125 35988855

[59] Varkhede N, Forrest ML. Understanding the monoclonal antibody disposition after subcutaneous administration using a minimal physiologically based pharmacokinetic model. J Pharm Pharmaceut Sci. 2018;21(1 Suppl):130s. 10.18433/jpps30028 PMC6613546 30011390

[60] Derakhshandeh R, Eshraghi J, Tavakolian M, Duffy KH, Veilleux J-C, Shi GH, et al. Performance characterization of spring-actuated prefilled pen devices for lebrikizumab and dupilumab. Expert Opin Drug Deliv. 2025;22(5):747–56. 40053331 10.1080/17425247.2025.2477656

[61] Derakhshandeh R, Meyers BA, Zhang J, Veilleux J-C, Shi GH, Vlachos PP. Subcutaneous depot formation and diffusion in autoinjector delivery: insights from high-speed synchrotron imaging. International Journal of Pharmaceutics. 2025;681:125804. 10.1016/j.ijpharm.2025.125804 40456425

[62] Struemper H, Murtaugh T, Gilbert J, Barton ME, Fire J, Groark J, et al. Relative bioavailability of a single dose of belimumab administered subcutaneously by prefilled syringe or autoinjector in healthy subjects. Clin Pharmacol Drug Dev. 2016;5(3):208–15. 27163500 10.1002/cpdd.219 PMC5063175

[63] Anumolu SS, Lindgren S, Vemula J, Floch D, Reynolds C, Wallny H-J, et al. Bioequivalence of canakinumab injected subcutaneously via an autoinjector device or a prefilled safety syringe device in healthy subjects. Clin Pharmacol Drug Dev. 2018;7(8):829–36. 29676864 10.1002/cpdd.455

[64] Zheng Y, Abuqayyas L, Megally A, Fuhr R, Sałapa K, Downie J, et al. Tezepelumab pharmacokinetics, safety, and tolerability after administration via vial-and-syringe, accessorized prefilled syringe, or autoinjector: a randomized trial in healthy volunteers. Clin Ther. 2021;43(1):142–55. 33380362 10.1016/j.clinthera.2020.11.014

[65] Doughty DV, Clawson CZ, Lambert W, Subramony JA. Understanding subcutaneous tissue pressure for engineering injection devices for large-volume protein delivery. J Pharm Sci. 2016;105(7):2105–13. 27287520 10.1016/j.xphs.2016.04.009

[66] Schneider A, Jost R, Jordi C, Lange J. Autoinjectors for large-volume subcutaneous drug delivery: a review of current research and future directions. Expert Opin Drug Deliv. 2023;20(6):815–30. 37272119 10.1080/17425247.2023.2219891

[67] Martinez MN. Factors influencing the use and interpretation of animal models in the development of parenteral drug delivery systems. AAPS J. 2011;13(4):632–49. 21971647 10.1208/s12248-011-9303-8 PMC3231859

[68] Sánchez-Félix M, Burke M, Chen HH, Patterson C, Mittal S. Predicting bioavailability of monoclonal antibodies after subcutaneous administration: open innovation challenge. Adv Drug Deliv Rev. 2020;167:66–77. 32473188 10.1016/j.addr.2020.05.009

[69] Purcell M, Babee S, Galluppi M, Cline J, Hu G, Petrescu I, et al. Characterization of large volume subcutaneous injections using computed tomography imaging and simultaneous pressure measurements. Front Drug Delivery. 2023;3:1223177. 10.3389/fddev.2023.1223177 PMC12363303 40838048

[70] Hou P, Zheng F, Corpstein CD, Xing L, Li T. Multiphysics modeling and simulation of subcutaneous injection and absorption of biotherapeutics: sensitivity analysis. Pharm Res. 2021;38(6):1011–30. 34080101 10.1007/s11095-021-03062-4

[71] Comley K, Fleck N. Deep penetration and liquid injection into adipose tissue. J Mech Mater Struct. 2011;6(1):127–40.

[72] Jackisch C, Müller V, Maintz C, Hell S, Ataseven B. Subcutaneous administration of monoclonal antibodies in oncology. Geburtshilfe Frauenheilkd. 2014;74(4):343. 25076790 10.1055/s-0034-1368173 PMC4078128

[73] Haller MF. Converting intravenous dosing to subcutaneous dosing with recombinant human hyaluronidase. Advanstar Communications Inc; 2007.

[74] Collins D, Kourtis L, Thyagarajapuram N, Sirkar R, Kapur S, Harrison M, et al. Optimizing the bioavailability of subcutaneously administered biotherapeutics through mechanochemical drivers. Pharm Res. 2017;34:2000–11. 28707164 10.1007/s11095-017-2229-9 PMC5579144

[75] US Food and Drug Administration. Draft guidance for industry: Bridging for drug-device and biologic-device combination products. 2019.

[76] Hu P, Wang J, Florian J, Shatzer K, Stevens AM, Gertz J, et al. Systematic review of device parameters and design of studies bridging biologic-device combination products using prefilled syringes and autoinjectors. AAPS J. 2020;22:1–9. 10.1208/s12248-020-0433-8 32107671

[77] de Lucio M, Bures M, Ardekani AM, Vlachos PP, Gomez H. Isogeometric analysis of subcutaneous injection of monoclonal antibodies. Comput Methods Appl Mech Eng. 2021;373:113550.

[78] Bittner B, Munoz J, Frank S, Dolton M, Ravanello R, Dembowski M, et al. Clinical qualification of subcutaneous injection devices for monoclonal antibodies. BioDrugs. 2026;40(1):5–21. 41247399 10.1007/s40259-025-00755-9 PMC12790514

[79] Gresham J, Bruin G, Picci M, Bechtold-Peters K, Dimke T, Davies E, et al. Visualisation and quantification of subcutaneous injections of different volumes, viscosities and injection rates: an ex-vivo micro-CT study. J Pharm Sci. 2024;113(12):3447–56. 39306036 10.1016/j.xphs.2024.08.019

[80] Mooney M. The viscosity of a concentrated suspension of spherical particles. J Colloid Sci. 1951;6(2):162–70.

[81] Ross PD, Minton AP. Hard quasispherical model for the viscosity of hemoglobin solutions. Biochem Biophys Res Commun. 1977;76(4):971–6. 20088 10.1016/0006-291x(77)90950-0

[82] Burckbuchler V, Mekhloufi G, Giteau AP, Grossiord J, Huille S, Agnely F. Rheological and syringeability properties of highly concentrated human polyclonal immunoglobulin solutions. Eur J Pharm Biopharm. 2010;76(3):351–6. 20719247 10.1016/j.ejpb.2010.08.002

[83] Binabaji E, Ma J, Zydney AL. Intermolecular interactions and the viscosity of highly concentrated monoclonal antibody solutions. Pharm Res. 2015;32:3102–9. 25832501 10.1007/s11095-015-1690-6

[84] Mach H, Gregory SM, Mackiewicz A, Mittal S, Lalloo A, Kirchmeier M, et al. Electrostatic interactions of monoclonal antibodies with subcutaneous tissue. Ther Deliv. 2011;2(6):727–36. 22822505 10.4155/tde.11.31

[85] Zou P. Predicting human bioavailability of subcutaneously administered fusion proteins and monoclonal antibodies using human intravenous clearance or antibody isoelectric point. AAPS J. 2023;25(3):31. 36959523 10.1208/s12248-023-00798-2

[86] Olsen SN, Andersen KB, Randolph TW, Carpenter JF, Westh P. Role of electrostatic repulsion on colloidal stability of bacillus halmapalus alpha-amylase. Biochem Biophys Acta (BBA)-Proteins Proteom. 2009;1794(7):1058–65. 10.1016/j.bbapap.2009.02.010 19281873

[87] Yadav S, Laue TM, Kalonia DS, Singh SN, Shire SJ. The influence of charge distribution on self-association and viscosity behavior of monoclonal antibody solutions. Mol Pharm. 2012;9(4):791–802. 22352470 10.1021/mp200566k

[88] Connolly BD, Petry C, Yadav S, Demeule B, Ciaccio N, Moore JM, et al. Weak interactions govern the viscosity of concentrated antibody solutions: high-throughput analysis using the diffusion interaction parameter. Biophys J. 2012;103(1):69–78. 22828333 10.1016/j.bpj.2012.04.047 PMC3388210

[89] Li C, Zhong X, Ardekani AM. Numerical studies of the lymphatic uptake rate. Computers in Biology and Medicine. 2023;165:107380. 10.1016/j.compbiomed.2023.107380 37634464

[90] Langmuir I. The adsorption of gases on plane surfaces of glass, mica and platinum. J Am Chem Soc. 1918;40(9):1361–403.

[91] Rabe M, Verdes D, Seeger S. Understanding protein adsorption phenomena at solid surfaces. Adv Coll Interface Sci. 2011;162(1–2):87–106. 10.1016/j.cis.2010.12.007 21295764

[92] Dang X, Shih H, Sharma R, Angwin-Kaerner DT, Lin K, Kapur S, et al. Clinical investigation of large volume subcutaneous delivery up to 25 ml for lean and non-lean subjects. Pharm Res. 2024;41(4):751–63. 38443633 10.1007/s11095-024-03683-5

[93] Mathaes R, Koulov A, Joerg S, Mahler H-C. Subcutaneous injection volume of biopharmaceuticals—pushing the boundaries. J Pharm Sci. 2016;105(8):2255–9. 27378678 10.1016/j.xphs.2016.05.029

[94] Styles IK, Feeney OM, Nguyen T-H, Brundel DH, Kang DW, Clift R, et al. Removal of interstitial hyaluronan with recombinant human hyaluronidase improves the systemic and lymphatic uptake of cetuximab in rats. J Control Release. 2019;315:85–96. 31655131 10.1016/j.jconrel.2019.10.040

[95] Dolton MJ, Chesterman A, Moein A, Sink KM, Waitz A, Blondeau K, et al. Safety, tolerability, and pharmacokinetics of high-volume subcutaneous crenezumab, with and without recombinant human hyaluronidase in healthy volunteers. Clin Pharmacol Therapeutics. 2021;110(5):1337–48. 10.1002/cpt.2385 34347883

[96] Mirrashed F, Sharp J, Krause V, Morgan J, Tomanek B. Pilot study of dermal and subcutaneous fat structures by MRI in individuals who differ in gender, BMI, and cellulite grading. Skin Res Technol. 2004;10(3):161–8. 10.1111/j.1600-0846.2004.00072.x 15225265

[97] Lancerotto L, Stecco C, Macchi V, Porzionato A, Stecco A, De Caro R. Layers of the abdominal wall: anatomical investigation of subcutaneous tissue and superficial fascia. Surg Radiol Anat. 2011;33:835–42. 21212951 10.1007/s00276-010-0772-8

[98] Causey L, Cowin SC, Weinbaum S. Quantitative model for predicting lymph formation and muscle compressibility in skeletal muscle during contraction and stretch. Proc Natl Acad Sci. 2012;109(23):9185–90. 22615376 10.1073/pnas.1206398109 PMC3384164

[99] Negrini D, Moriondo A, Mukenge S. Transmural pressure during cardiogenic oscillations in rodent diaphragmatic lymphatic vessels. Lymphat Res Biol. 2004;2(2):69–81. 15615488 10.1089/lrb.2004.2.69

[100] Haynes FW. Factors which influence the flow and protein content of subcutaneous lymph in the dog: II. the effect of certain substances which alter the capillary circulation. Am J Physiol Legacy Content. 1932;101(4):612–20.

[101] Engeset A, Olszewski W, Jaeger P, Sokolowski J, Theodorsen L. Twenty-four hour variation in flow and composition of leg lymph in normal men. Acta Physiol Scand. 1977;99(2):140–8. 842370 10.1111/j.1748-1716.1977.tb10364.x

[102] McGeown J, McHale N, Thornbury K. The role of external compression and movement in lymph propulsion in the sheep hind limb. J Physiol. 1987;387(1):83–93. 3656186 10.1113/jphysiol.1987.sp016564 PMC1192495

[103] Lane K, Worsley D, McKenzie D. Exercise and the lymphatic system: implications for breast-cancer survivors. Sports Med. 2005;35:461–71. 15974632 10.2165/00007256-200535060-00001

[104] Jain SM, Pandey K, Lahoti A, Rao PK. Evaluation of skin and subcutaneous tissue thickness at insulin injection sites in Indian, insulin naïve, type-2 diabetic adult population. Indian J Endocrinol Metabolism. 2013;17(5):864–70. 10.4103/2230-8210.117249 PMC3784870 24083168

[105] Uren RF, Howman-Giles R, Thompson JF. Variation in cutaneous lymphatic flow rates. Ann Surg Oncol. 1997;4(3):279–80. 9142392 10.1007/BF02306624

[106] Uren R, Howman-Giles R, Thompson J, Roberts J, Bernard E. Variability of cutaneous lymphatic flow rates in melanoma patients. Melanoma Res. 1998;8(3):279–82. 9664151 10.1097/00008390-199806000-00012

[107] Akhras V, Stanton AW, Levick JR, Mortimer PS. A quantitative examination of lymph drainage from perilesion skin in human melanoma. Lymphat Res Biol. 2012;10(3):107–11. 22984906 10.1089/lrb.2012.0009

[108] Hollander W, Reilly P, Burrows B, et al. Lymphatic flow in human subjects as indicated by the disappearance of i-labeled albumin from the subcutaneous tissue. J Clin Investig. 1961;40(2):222–33. 10.1172/JCI104248 PMC290714 13715314

[109] Pain SJ, Nicholas RS, Barber RW, Ballinger JR, Purushotham AD, Mortimer PS, et al. Quantification of lymphatic function for investigation of lymphedema: depot clearance and rate of appearance of soluble macromolecules in blood. J Nucl Med. 2002;43(3):318–24. 11884490

[110] Karlsen TV, McCormack E, Mujic M, Tenstad O, Wiig H. Minimally invasive quantification of lymph flow in mice and rats by imaging depot clearance of near-infrared albumin. Am J Physiol Heart Circul Physiol. 2012;302(2):H391–401. 10.1152/ajpheart.00842.2011 22101523

[111] Zheng F, Hou P, Corpstein CD, Xing L, Li T. Multiphysics modeling and simulation of subcutaneous injection and absorption of biotherapeutics: model development. Pharm Res. 2021;38:607–24. 33811278 10.1007/s11095-021-03032-w

[112] Han D, Rahimi E, Aramideh S, Ardekani AM. Transport and lymphatic uptake of biotherapeutics through subcutaneous injection. J Pharm Sci. 2022;111(3):752–68. 34624293 10.1016/j.xphs.2021.09.045

[113] Han D, Li C, Araimdeh S, Sree V, Rahimi E, Tepole AB, et al. Lymphatic uptake of biotherapeutics through a 3D hybrid discrete-continuum vessel network in the skin tissue. J Control Release. 2023;354:869–88. 10.1016/j.jconrel.2022.12.045 36634711

[114] Leng Y, de Lucio M, Gomez H. Using poro-elasticity to model the large deformation of tissue during subcutaneous injection. Comput Methods Appl Mech Eng. 2021;384:113919.

[115] de Lucio M, Leng Y, Hans A, Bilionis I, Brindise M, Ardekani AM, et al. Modeling large-volume subcutaneous injection of monoclonal antibodies with anisotropic porohyperelastic models and data-driven tissue layer geometries. J Mech Behav Biomed Mater. 2023;138:105602. 36529050 10.1016/j.jmbbm.2022.105602

[116] de Lucio M, Leng Y, Wang H, Ardekani AM, Vlachos PP, Shi G, et al. Computational modeling of the effect of skin pinch and stretch on subcutaneous injection of monoclonal antibodies using autoinjector devices. Biomech Model Mechanobiol. 2023;22(6):1965–82. 37526775 10.1007/s10237-023-01746-x

[117] Leng Y, Ardekani AM, Gomez H. A poro-viscoelastic model for the subcutaneous injection of monoclonal antibodies. J Mech Phys Solids. 2021;155:104537.

[118] Han D, Huang Z, Rahimi E, Ardekani AM. Solute transport across the lymphatic vasculature in a soft skin tissue. Biology. 2023;12(7):942. 10.3390/biology12070942 PMC10375963 37508373

[119] Li C, Zhong X, Rahimi E, Ardekani AM. A multi-scale numerical study of monoclonal antibodies uptake by initial lymphatics after subcutaneous injection. Int J Pharm. 2024;661:124419. 10.1016/j.ijpharm.2024.124419 38972522

[120] de Lucio M, Leng Y, Wang H, Vlachos PP, Gomez H. Modeling drug transport and absorption in subcutaneous injection of monoclonal antibodies: Impact of tissue deformation, devices, and physiology. Int J Pharm. 2024;661:124446. 10.1016/j.ijpharm.2024.124446 38996825

